# Implementing evidence-based practices in rural settings: a scoping review of theories, models, and frameworks

**DOI:** 10.3389/frhs.2024.1326777

**Published:** 2024-07-05

**Authors:** Robert E. Brady, Kathleen D. Lyons, Courtney J. Stevens, Cassandra M. Godzik, Andrew J. Smith, Pamela J. Bagley, Elaina J. Vitale, Steven L. Bernstein

**Affiliations:** ^1^ Department of Psychiatry, Dartmouth-Hitchcock Medical Center, Lebanon, NH, United States; ^2^ Geisel School of Medicine at Dartmouth, Hanover, NH, United States; ^3^ Department of Occupational Medicine, Massachusetts General Hospital Institute of Health Professions, Boston, MA, United States; ^4^ Lyda Hill Institute for Human Resilience, University of Colorado, Colorado Springs, Colorado Springs, CO, United States; ^5^ Biomedical Libraries, Dartmouth College, Hanover, NH, United States; ^6^ Department of Emergency Medicine, Dartmouth-Hitchcock Medical Center, Lebanon, NH, United States

**Keywords:** scoping review, implementation frameworks, re-aim, CFIR, CBPR, iPARIHS

## Abstract

**Background:**

Rural healthcare has unique characteristics that affect the dissemination and implementation of evidence-based interventions. Numerous theories, models, and frameworks have been developed to guide implementation of healthcare interventions, though not specific to rural healthcare. The present scoping review sought to identify the theories, models, and frameworks most frequently applied to rural health and propose an approach to rural health research that harnesses selected constructs from these theories, models, and frameworks. This resulting synthesis can serve as a guide to researchers, policy makers, and clinicians seeking to employ commonly used theories, models, and frameworks to rural health.

**Methods:**

We used the Scopus abstract indexing service to identify peer-reviewed literature citing one or more of theories, models, or frameworks used in dissemination and implementation research and including the word “rural” in the Title, Abstract, or Keywords. We screened the remaining titles and abstracts to ensure articles met additional inclusion criteria. We conducted a full review of the resulting 172 articles to ensure they identified one or more discrete theory, model, or framework applied to research or quality improvement projects. We extracted the theories, models, and frameworks and categorized these as process models, determinant frameworks, classic theories, or evaluation frameworks.

**Results:**

We retained 61 articles of which 28 used RE-AIM, 11 used Community-Based Participatory Research (CBPR) framework, eight used the Consolidated Framework for Implementation Research (CFIR), and six used the integrated-Promoting Action on Research Implementation in Health Services (iPARIHS). Additional theories, models, and frameworks were cited in three or fewer reports in the literature. The 14 theories, models, and frameworks cited in the literature were categorized as seven process models, four determinant frameworks, one evaluation framework, and one classic theory.

**Conclusions:**

The RE-AIM framework was the most frequently cited framework in the rural health literature, followed by CBPR, CFIR, and iPARIHS. A notable advantage of RE-AIM in rural healthcare settings is the focus on reach as a specified outcome, given the challenges of engaging a geographically diffuse and often isolated population. We present a rationale for combining the strengths of these theories, models, and frameworks to guide a research agenda specific to rural healthcare research.

**Systematic Review Registration:**

https://osf.io/fn2cd/.

## 1 Introduction

Rural settings are characterized by features such as low population density and geographic isolation (1). The population of people living in rural settings in the United States experience worsened health outcomes compared to densely populated urban settings (2). The causes of these disparities are numerous and complexly intertwined, leading to what has been termed the “rural mortality penalty.” (3) For example, many rural settings have less access to primary and specialty medical care, increased travel time for urgent and emergency care, and more workforce shortages than urban settings (4–6). As such, disseminating and implementing evidence-based healthcare practices and policies into rural settings includes unique challenges across a range of contextual variables (7).

Implementation science has the potential to address the challenges of healthcare delivery in rural settings (8). Among the most significant developments from this young but rapidly maturing field is the development and refinement of dissemination (i.e., spreading knowledge of an innovation or intervention) and implementation (i.e., getting the innovation or intervention into routine practice) theories, models, and frameworks used to guide the design, implementation, and evaluation of evidence-based interventions. The definitions of theories, models, and frameworks and their distinctions within dissemination and implementation research was most effectively provided by Nilsen (9). In brief, theories seek to explain observed phenomena, models seek to simplify otherwise complex observed or predicted phenomena, and frameworks seek to establish a structure for the relation between components of those phenomena. There are currently at least 110 published theories, models, and frameworks (10), expanded from the seminal paper by Tabak and colleagues that identified 61 such theories, models, and frameworks (11). Together, these compilations provide a convenient source for identifying and selecting theories, models, and frameworks that can guide implementation, determination of factors that can influence implementation, or evaluation and interpretation of implementation outcomes (9).

Our overarching research goal is to improve understanding of implementation methods and approaches in rural healthcare to support the development of a strategic and cohesive approach to conducting evidence-based healthcare throughout rural-based healthcare delivery systems. Our first step in this process was to conduct a scoping review of healthcare dissemination and implementation studies occurring in rural settings. Scoping reviews are appropriate for providing an overview of a topic (e.g., what do we know about implementing evidence-based practices in rural settings?), as opposed to answering a tightly worded clinical question (e.g., is palliative care effective for rural-dwelling patients recently diagnosed with advanced cancer?) (12). The results of this scoping review will inform model development for the integration of research and practice within rural academic medical systems.

The specific aim of this scoping review is to characterize the theories, models, and frameworks used in rural healthcare research and quality improvement including the frequency and type of theory, model, or framework that have been applied in rural settings to support the development of a strategic and cohesive approach to conducting evidence-based healthcare throughout rural-based healthcare delivery systems. We limited this review to research that examines evidence-based medical interventions implemented and delivered within rural settings, including in the context of quality improvement initiatives. Thus, the review does not include those studies that would examine themes and constructs considered preparatory for research, such as formative evaluations, case studies of interventions, and observational studies examining a clinical phenomenon (e.g., survey designs and secondary data analyses).

## 2 Methods

We used the steps recommended by Arksey and O'Malley for conducting scoping reviews. We pilot tested the search strategy and operationalization of the eligibility criteria prior to conducting the full search (13). The steps of the scoping review were as follows: (1) specify the research question(s), (2) identify the relevant studies, (3) select studies using predetermined inclusion/exclusion criteria, (4) extract data, and (5) synthesize and report the results. Our protocol was registered in the Open Science Forum prior to extracting data (https://osf.io/fn2cd). We used the Preferred Reporting Items for Systematic Reviews and Meta-Analyses extension for Scoping Reviews Checklist to ensure best practices in this review (See Supplementary File S1).

### 2.1 Research question

Given the overarching goal of improving understanding of implementation methods and approaches in rural healthcare, we focused on this broad question for the present scoping review: What dissemination and implementation theories, models, and frameworks are used in rural healthcare research?

### 2.2 Identifying relevant studies

We partnered with librarians at our institution to create a rigorous search strategy. The librarians first identified the primary citations for each of the 110 theories, models, and frameworks catalogued on the *Dissemination and Implementation Models in Health* website (www.dissemination-implementation.org) (10). The librarians constructed a search string within the Scopus (Elsevier) database for all sources citing these articles using the REFTITLE field. To focus on rural settings, they searched Scopus, a large interdisciplinary database that includes records from Medline and EMBASE, for the term “rural” in the TITLE, ABSTRACT, or KEYWORD fields and combine these results with the sources citing dissemination and implementation theories, models, and frameworks. This created a citation list consisting of all articles published between 2022 and 1998, the latter being a proposed initial date of formal implementation science research coinciding with the formation of Quality Enhancement Research Initiative (QUERI). This search strategy yielded 1,677 publications. The search was conducted on February 2, 2022. The complete search strategy is available in Supplementary File S2. The publications were loaded into the Covidence software program to manage the screening and extraction process (14).

### 2.3 Selecting studies

We used the following inclusion criteria:

1) Presents data (qualitative or quantitative) from a trial of an intervention or quality improvement effort to improve delivery of an intervention within the physical medicine, mental health, or pharmacy literature. Rationale: For study protocol publications that meet this criterion, we searched for available outcomes papers for inclusion. We determined this *a priori* based on the observation that many protocol papers specify a theory, model, or framework to be used in the study, but do not necessarily report this in the outcomes paper. Protocol papers that did not have a corresponding outcomes paper were removed prior to data extraction. Additionally, we used a broad definition of healthcare settings for this review to reflect the variability of settings in which healthcare is delivered in rural communities. We included studies or projects in which an intervention was delivered for the explicit purpose of modifying a health outcome regardless of the regular use physical location (e.g., community resource centers, churches, schools) within the physical medicine, mental health, or pharmacy domains. This also included traditional healthcare settings such as community health centers and rural hospitals.
2) Reports the use of a discrete theory, model, or framework included in the *Dissemination and Implementation Models in Health* registry. Rationale: Because our goal is to develop or tailor a model that will guide our research and practice, we sought articles that explicitly locate themselves within implementation science and use a theory, model, or framework to guide their study or quality improvement design.
3) Includes the word “rural” in the title or abstract or author keywords. Rationale: There are many operational definitions of rurality and many studies inconsistently report their definition of rurality. We chose to be inclusive, and included any study where the authors identify their setting or population as rural within the title, abstract, or keywords.
4) Reports on data collected within the United States and published in English. Rationale: We recognize that many high-quality studies occur outside the United States, but also that the U.S. population represents a broad range of cultural, healthcare, and demographic variables that make it unique relative to much of the world. Additionally, studies situated in low- and middle-income countries have infrastructure and cultural expectations that result in conclusions less germane to the approaches used in this system.
5) Published after 1998, coinciding with the initiation of the QUERI. Rationale: Although dissemination and implementation theories, models, and frameworks existed before this date, the initiation of QUERI was selected as an early point of formal research on the dissemination and implementation of interventions in healthcare specifically (15).

**Figure 1:**
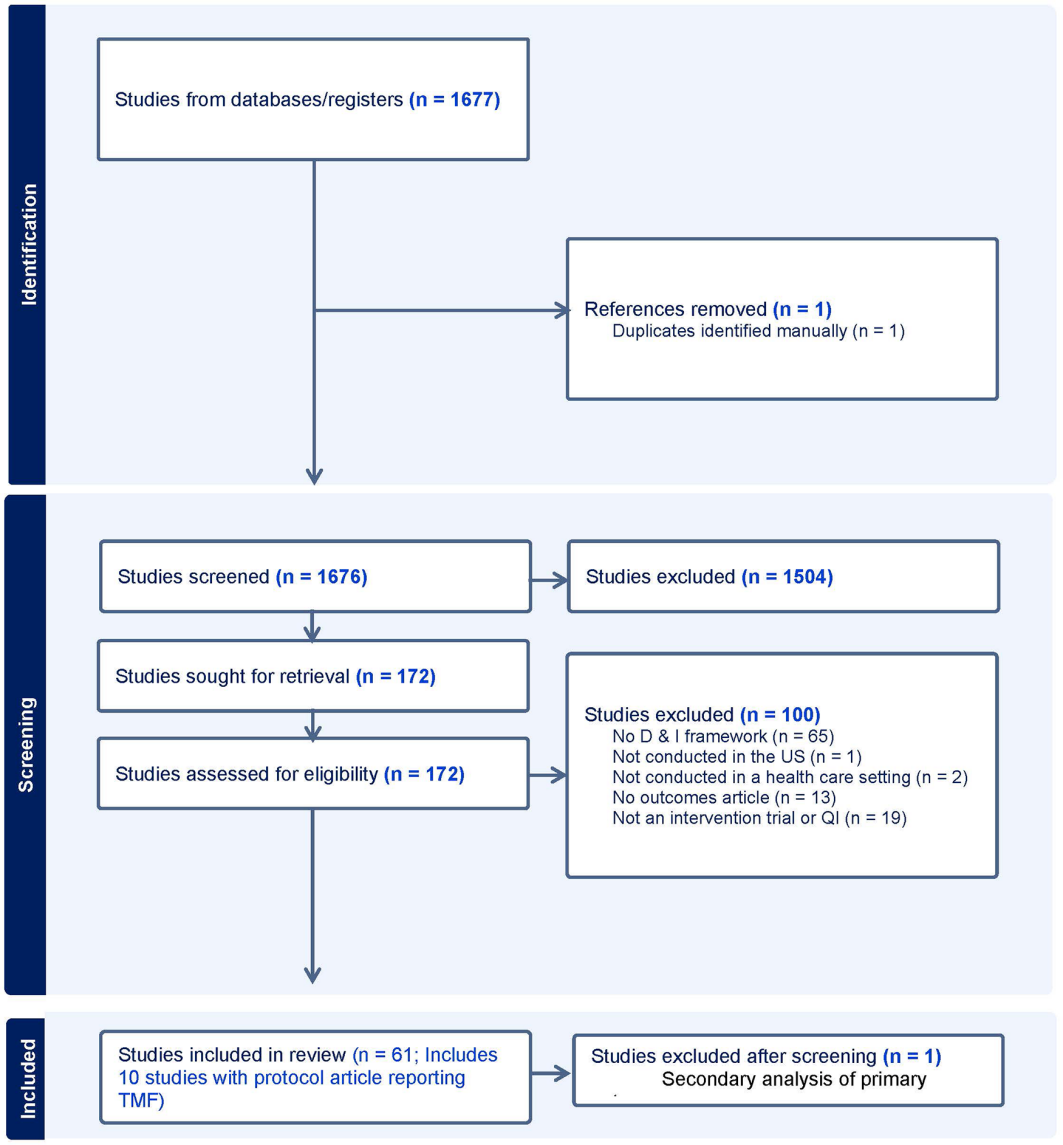
Flow diagram of search strategy and article selection.

The study team conducted an initial pilot test of the review method using a sample of 50 randomly selected citations prior to the full review. The pilot search verified that the search terms applied to the specified databases were yielding the expected articles, namely those identifying research citing dissemination and implementation theories, models, or frameworks applied to rural health research. This also provided a training set for the study abstract screening process. A team of four reviewers (RB, CS, CG, AS) screened each citation to eliminate articles that did not meet the inclusion criteria. The first stage of screening utilized the title and abstract and the second stage of screening utilized the full text of each article. In both stages, each citation was screened by two independent screeners unaware of the other screener's vote for inclusion or exclusion of the citation. A third screener (RB or KL) provided a tie-breaking vote reviewed for citations with conflicting votes for inclusion or exclusion. For articles where it was unclear whether the authors applied a theory, model, or framework to the design or execution of the study, the first author (RB) contacted the corresponding author for confirmation. A full report of these data is presented in Figure 1.

### 2.4 Data extraction

We constructed a data extraction matrix in Covidence to chart and sort the data (14). The matrix included basic information about each study (author, year, title, and publication date) and the theory, model, or framework used in the study. Extraction was completed by two team members per study. Pairs met after initial extraction to resolve disagreements in a consensus process moderated by the first author (RB).

### 2.5 Data synthesis and reporting

We calculated the frequencies of each theory, model, or framework and categorized the year of publication by decade. Using the definitions posed by Nilsen, we then categorized each as a (a) classic theory (i.e., theories that originate outside of implementation science specifically, but are used to understand implementation processes), (b) process model (i.e., that guide or describe the how evidence is translated into practice) (c) determinants framework or theory (i.e., explain factors that influence implementation) or (d) evaluation framework (i.e., guide the measurement of implementation outcomes) (9).

## 3 Results

### 3.1 Study selection

A total of 1,683 candidate publications were identified from the search strategy. The study team completed a title and abstract screening process that resulted in the exclusion of 1,508 publications that failed to meet inclusion criteria. Data collected outside of the United States was the most frequent reason for exclusion. The full text review excluded an additional 100 articles that did not meet eligibility criteria, including four in which the corresponding author confirmed that a cited theory, model, or framework was not actually applied to the design or conduct of the trial. Of the remaining 72 articles, 10 were protocol articles for which there was an outcomes article signifying that the study had be conducted. For review and charting purposes, these studies were combined with the outcomes article such that if the protocol paper described the theory, model, or framework used, but the outcomes article did not, it was still identified as used for the purpose of the study reported in the outcomes article. One article by Zoellner and colleagues provided an additional analysis of a single trial, which was subsequently removed to reduce inflation of the corresponding framework's use, leaving a final sample of 61 articles. The Preferred Reporting Items for Systematic Reviews and Meta-Analyses (PRISMA) flow diagram depicts our study selection process in Figure 1 (16). All citations for the included articles are provided in Table 1.

**Table 1:** Characteristics of included studies.

First author (year)	Title	TMF	TMF category
Allicock (2017) (17)	Peer Connect for African American breast cancer survivors and caregivers: a train-the-trainer approach for peer support	DoI	Classic Theory
Ard (2017) (18)	Weight loss and improved metabolic outcomes amongst rural African American women in the Deep South: six-month outcomes from a community-based randomized trial	CBPR	Process Model
Balis (2018) (19)	Beginning with the end in mind: Contextual considerations for scaling-out a community-based intervention	RE-AIM	Evaluation Framework
Baloh (2018) (20)	Implementing team huddles in small rural hospitals: How does the Kotter model of change apply?	Kotter model of change	Process Model
Baloh (2018) (21)	Types of internal facilitation activities in hospitals implementing evidence-based interventions	iPARIHS	Determinant Framework
Befort (2021) (22)	Effect of Behavioral Therapy with In-Clinic or Telephone Group Visits vs In-Clinic Individual Visits on Weight Loss among Patients with Obesity in Rural Clinical Practice: A Randomized Clinical Trial	RE-AIM	Evaluation Framework
Bender (2021) (23)	The Asthma Toolkit Bootcamp to Improve Rural Primary Care for Pediatric Asthma	RE-AIM	Evaluation Framework
Bernstein (2009) (24)	A preliminary report of knowledge translation: Lessons from taking screening and brief intervention techniques from the research setting into regional systems of care	RE-AIM	Evaluation Framework
Billue (2012) (25)	Medication intensification in diabetes in rural primary care: A cluster-randomized effectiveness trial	RE-AIM	Evaluation Framework
Bittar (2018) (26)	Implementation of a standardized data- collection system for comprehensive appraisal of cleft care	RE-AIM	Evaluation Framework
Brunet (2022) (27)	Increasing buprenorphine access for veterans with opioid use disorder in rural clinics using telemedicine	CFIR	Determinant Framework
Carman (2015) (28)	Organizational Variation in Implementation of an Evidence-Based Human Papillomavirus Intervention	iPARIHS	Determinant Framework
Cené (2017) (29)	A multicomponent quality improvement intervention to improve blood pressure and reduce racial disparities in rural primary care practices	iPARIHS	Determinant Framework
Chambers (2021) (30)	Empowering Native Adolescents: Responsibility for Their Health Behaviors	CBPR	Process Model
Cicutto (2014) (31)	Improving asthma care in rural primary care practices: A performance improvement project	PRISM	Combined Framework
Conway (2016) (32)	Rural health networks and care coordination: Health care innovation in frontier communities to improve patient outcomes and reduce health	CBPR	Process Model
Eaker (2001) (33)	Women's health alliance intervention study: Increasing community breast and cervical cancer screening	CBPR	Process Model
Earp (2002) (34)	Increasing use of mammography among older, rural African American women: Results from a community trial	RE-AIM	Evaluation Framework
Fiallo-Scharer (2019) (35)	Impact of family-centered tailoring of pediatric diabetes self-management resources	RE-AIM	Evaluation Framework
Fisher (2018) (36)	Adaptation and Implementation of a Transitional Care Protocol for Patients Undergoing Complex Abdominal Surgery	REP	Process Model
Garney (2016) (37)	Using an Interactive Systems Framework to Expand Telepsychology Innovations in Underserved Communities	ISF	Determinant Framework
Garvin (2021) (38)	Use of Video Telehealth Tablets to Increase Access for Veterans Experiencing Homelessness	RE-AIM	Evaluation Framework
Gilmartin (2022) (39)	Effectiveness of the rural transitions nurse program for Veterans: A multicenter implementation study	RE-AIM; PRISM	Evaluation Framework; Combined Framework
Harry (2020) (40)	Pre-implementation adaptation of primary care cancer prevention clinical decision support in a predominantly rural healthcare system	CFIR	Determinant Framework
Harry (2022) (41)	Human Papillomavirus vaccination clinical decision support for young adults in an upper midwestern healthcare system: a clinic cluster-randomized control trial	RE-AIM; CFIR	Evaluation Framework; Determinant Framework
Hirko (2021) (42)	Implementation of physical activity programs for rural cancer survivors: Challenges and opportunities	RE-AIM	Evaluation Framework
Johansson (2019) (43)	Adapting an Evidence-based Cardiovascular Disease Risk Reduction Intervention to Rural Communities	RE-AIM	Evaluation Framework
Kempe (2013) (44)	Population-based versus practice-based recall for childhood immunizations: A randomized controlled comparative effectiveness trial	RE-AIM	Evaluation Framework
Kennedy (2014) (45)	People United to Sustain Health (PUSH): A Community-Based Participatory Research Study	CBPR	Process Model
Kobe (2022) (46)	Implementation of an Intensive Telehealth Intervention for Rural Patients with Clinic-Refractory Diabetes	RE-AIM	Evaluation Framework
Koenig (2016) (47)	Pre-Implementation Strategies to Adapt and Implement a Veteran Peer Coaching Intervention to Improve Mental Health Treatment Engagement Among Rural Veterans	PARIHS	Determinant Framework
Kolb (2021) (48)	Implementation of clinical practice guidelines for low back pain: A case control cohort study of knowledge translation in a multi-site healthcare organization	RE-AIM	Evaluation Framework
Komro (2017) (49)	Multilevel prevention trial of alcohol use among American Indian and white high school students in the Cherokee nation	CBPR	Process Model
Lin (2016) (50)	Using the 4 pillars^TM^ practice transformation program to increase adult influenza vaccination and reduce missed opportunities in a randomized cluster trial	Active Implementation Framework; DoI	Determinant Framework; Classic Theory
McCarthy (2021) (51)	Understanding adaptations in the Veteran Health Administration's Transitions Nurse Program: refining methodology and pragmatic implications for scale-up	PRISM; RE-AIM	Combined Framework; Evaluation Framework
McCullough (2021) (52)	Introducing clinical pharmacy specialists into interprofessional primary care teams Assessing pharmacists’ team integration and access to care for rural patients	RE-AIM	Evaluation Framework
Modica (2019) (53)	Colorectal Cancer: Applying the Value Transformation Framework to increase the percent of patients receiving screening in Federally Qualified Health Centers	Framework for Spread	Process Model
Mudderman (2020) (54)	The Effect of an Evidence-Based Practice Education and Mentoring Program on Increasing Knowledge, Practice, and Attitudes Toward Evidence-Based Practice in a Rural Critical Access Hospital	Iowa Model of Evidence-Based Practice	Process Model
Nápoles (2020) (55)	Nuevo Amanecer-II: Results of a randomized controlled trial of a community-based participatory, peer-delivered stress management intervention for rural Latina breast cancer survivors	CBPR; Transcreation Framework	Process Model
Naik (2019) (56)	Effect of Telephone-Delivered Collaborative Goal Setting and Behavioral Activation vs Enhanced Usual Care for Depression Among Adults With Uncontrolled Diabetes: A Randomized Clinical Trial	RE-AIM	Evaluation Framework
Olson (2008) (57)	Changing Adolescent Health Behaviors. The Healthy Teens Counseling Approach	RE-AIM	Evaluation Framework
Palm (2020) (58)	An initiative to implement immediate postpartum long-acting reversible contraception in rural New Mexico	CFIR	Determinant Framework
Porter (2019) (59)	SIPsmartER delivered through rural, local health districts: Adoption and implementation outcomes	RE-AIM; ISF	Evaluation Framework; Determinant Framework
Porter (2021) (60)	Reach outcomes and costs of different physician referral strategies for a weight management program among rural primary care patients: Type 3 hybrid effectiveness-implementation trial	RE-AIM	Evaluation Framework
Powell (2005) (61)	Increasing mammography screening among African American women in rural areas	CBPR	Process Model
Powers (2018) (62)	Reaching out to rural caregivers and veterans with dementia utilizing clinical video-telehealth	iPARIHS	Determinant Framework
Preston (2018) (63)	Colorectal cancer screening in rural and poor-resourced communities	CBPR	Process Model
Samia (2014) (64)	The Maine Savvy Caregiver Project: Translating an Evidence-Based Dementia Family Caregiver Program Within the RE-AIM Framework	RE-AIM	Evaluation Framework
Schwartz (2013) (65)	Family-based risk reduction of obesity and metabolic syndrome: An overview and outcomes of the Idaho partnership for hispanic health	CBPR	Process Model
Snell-Rood (2019) (66)	Building interventions when distress is under debate: a case study from Appalachia	REP	Process Model
Snyder (2021) (67)	Rapid Adoption of Low-Threshold Buprenorphine Treatment at California Emergency Departments Participating in the CA Bridge Program	RE-AIM	Evaluation Framework
Sterba (2020) (68)	Determinants of evidence-based practice uptake in rural intensive care units a mixed methods study	EPIS; 4Es process theory	Combined Framework; Process Model
Taboada (2021) (69)	Implementing Goal Mama: Barriers and Facilitators to Introducing Mobile Health Technology in a Public Health Nurse Home-Visiting Program	CFIR	Determinant Framework
Thomson (2014) (70)	Delta Healthy Sprouts: A randomized comparative effectiveness trial to promote maternal weight control and reduce childhood obesity in the Mississippi Delta	RE-AIM	Evaluation Framework
Tinc (2020) (71)	Longitudinal use of the consolidated framework for implementation research to evaluate the creation of a rural center of excellence in transgender health	CFIR	Determinant Framework
Ward (2006) (72)	The impact of cancer coalitions on the dissemination of colorectal cancer materials to community organizations in rural appalachia	CBPR	Process Model
Ward (2017) (73)	Promoting Action on Research Implementation in Health Services framework applied to TeamSTEPPS implementation in small rural hospitals	iPARIHS	Determinant Framework
Ward (2021) (74)	Establishing clinical swallowing assessment services via telepractice: a multisite implementation evaluation	CFIR	Determinant Framework
Young (2018) (75)	Intensive Referral of Veterans to Mutual-Help Groups: A Mixed-Methods Implementation Evaluation	RE-AIM	Evaluation Framework
Zoellner (2016) (76)	Effects of a behavioral and health literacy intervention to reduce sugar-sweetened beverages: A randomized-controlled trial	RE-AIM	Evaluation Framework
Zulman (2019) (77)	Making connections: Nationwide implementation of video telehealth tablets to address access barriers in veterans	RE-AIM; CFIR	Evaluation Framework; Determinant Framework

CBPR, community based participatory research; CFIR, consolidated framework for implementation research; DoI, diffusion of innovations; EPIS, exploration participation implementation sustainment framework; iPARIHS, integrated-promoting action on research implementation in health services; ISF, interactive systems framework; PRISM, practical robust implementation and sustainability model; RE-AIM, reach effectiveness adoption implementation and maintenance framework; REP, replicating effective programs; TMF, theories models and frameworks.

### 3.2 Study characteristics

The 61 publications that were ultimately included in this scoping review depict a wide range of designs, including randomized and quasi-experimental clinical trials and quality improvement projects. All studies depicted efforts to implement or disseminate a healthcare intervention targeting physical health (*n* = 52) or mental health (including substance use) (*n* = 9). Publication dates ranged from 1998 to 2022, consistent with our use of the creation of QUERI as a start date for formal implementation research. We were unable to identify any publications from 1998 to 1999. Six studies were published in the 2000s, with the first study published in 2001. Thirty-three studies were published between 2010 and 2019, with the remaining 22 published since 2020.

### 3.3 Dissemination and implementation theories, models, and frameworks

Because our search strategy began with identifying articles that cited at least one validated and published implementation or dissemination theory, model, or framework, all publications included at least one theory, model, or framework. Some publications not included in the final review cited a theory, model, or framework, but did not indicate its use in the design, execution, or evaluation of outcomes. For example, several studies referenced a theory, model, or framework in the discussion section when describing a future direction of study. Those articles that did not indicate that the theory, model, or framework was used in the design or execution of the research or quality improvement or evaluation of its outcomes were excluded. Although RE-AIM and the Practical, Robust Implementation and Sustainability Model (78) (PRISM) are derived from the same underlying theory and are frequently used in tandem as partner frameworks where PRISM uses the outcomes outlined by RE-AIM, we examined the use of these frameworks separately in the literature to understand better how they are applied to the rural healthcare setting.

The most commonly used theories, models, and frameworks in the scope of this review were RE-AIM (79) (*n* = 28) and Community-Based Participatory Research framework (CBPR) (80) (*n* = 11). Less frequently cited were the integrated-Promoting Action on Research Implementation in Health Services (iPARIHS) (81) (*n* = 6) and Consolidated Framework for Implementation Research (CFIR) (82) (*n* = 8). PRISM was cited three times, either independently or in concert with RE-AIM. The Interactive Systems Framework (83), Replicating Effective Programs framework (84), and Diffusion of Innovations (85) were each cited two times. The remaining cited theories, models, and frameworks appeared once in the reviewed literature, including the Iowa Model of Evidence-Based Practice (86), Active Implementation Framework (87), Framework for Spread (88), 4Es Process Theory (89), and Exploration, Preparation, Implementation, Sustainment (EPIS) framework (90). The Kotter Model of Change (91) was not included in the larger collection of theories, models, and frameworks, but is relevant to rural health implementation and was included for its single citation. The use of each theory, model, or framework is presented in Table 1.

We categorized the 14 theories, models, and frameworks identified in this review according to the conventions outlined by Nilsen (9). This resulted in categorization of seven process models (CBPR, Iowa Model of Evidence-Based Practice, Replicating Effective Programs, Transcreation Framework, 4Es process theory, Framework for Spread, and Kotter Model of Change), four determinant frameworks (CFIR, iPARIHS, Interactive Systems Framework, and Active Implementation Framework), one classic theory (Diffusion of Innovations), and one evaluation framework (RE-AIM). Because the PRISM model is informed by elements from the IHI Model for Improvement, iPARIHS framework, and RE-AIM framework (78) and the EPIS framework includes elements of process and determinant frameworks (92) and do not clearly fit within a single category, we denoted an additional category of “combined” occurring four times. Category labels are included per citation in Table 1. The total frequency and citations per theory, model, or framework category are summarized in Table 2.

**Table 2:** Frequency of theories, models, and frameworks by nilsen categories.

Frequency of TMF category		
TMF category	TMF total Per category	TMF category citation frequency
Classical theory	1	2
Process model	7	17
Determinant framework	4	17
Evaluation framework	1	28
Combined framework	1	4

TMF, theories models frameworks

Finally, we assessed the number of theories, models, and frameworks used in each report. No citation reported using more than two and the majority (87%) cited a single theory, model, or framework. Among the theories, models, and frameworks used alongside another, RE-AIM was cited most, occurring in five of eight instances, alongside CFIR (2), PRISM (2), and ISF (1). The other reports used CBPR and the Transcreation Framework, EPIS and the 4Es process theory, or the Active Implementation Framework and Diffusion of Innovations.

## 4 Discussion

The specific aim of this scoping review was to identify the leading dissemination and implementation theories, models, and frameworks used to assess the impact of medical, psychosocial, and pharmacologic interventions in rural healthcare in the U.S, with the larger goal of advancing a research agenda that harnesses the leading implementation theories, models, and frameworks to guide efforts to improve the health and well-being of rural populations, where disparities in health outcomes and research remain present despite the growth of rural health as a focus of public policy (93).

The overwhelming majority of studies identified in this review specified use of RE-AIM (79) as the chosen framework to guide selection of outcomes. This is consistent with its overall prominence as a guiding framework for implementation and dissemination outcomes in healthcare research and delivery. Its frequent appearance in the reviewed literature likely reflects the premium placed on the identification of implementation outcomes in the larger dissemination and implementation research domain. Within the rural healthcare research domain, like other healthcare areas, RE-AIM has a particularly robust role in evaluating whether an intervention supported by implementation strategies achieves greater reach and adoption among rural healthcare facilities.

PRISM (78) was used in tandem with RE-AIM to aid in the determination of strategies to improve key implementation outcomes such as sustainment, but in relatively few studies, suggesting greater emphasis on the use of RE-AIM for defining outcomes. The specification of outcomes may also drive overall implementation, as attention to outcomes necessitates use of approaches and strategies focused on achieving those same outcomes. Given that reach among rural people is a specific challenge of rural health implementation, prioritizing the reach outcome from RE-AIM may be an optimal use of that framework. This does not suggest that the other domains within RE-AIM are irrelevant to rural health, but rather that emphasizing reach given the recognized difficulties getting into these populations has particular importance for advancing rural health.

The frequent appearance of CBPR (80) in the studies included in this review is expected given that research teams developing and evaluating interventions for deployment in rural settings are often not co-located in these communities. As a process model useful in rural health, CBPR has relevance in increasing engagement in the innovation and evaluation in a research context. Indeed, many clinical trials are at least initially launched by academic institutions that are not deeply engaged with the rural community structure, which then may hesitate to make substantive changes to settings (e.g., modifying clinical infrastructure) with their own culture and way of delivering care. Prioritizing the use of CBPR as a guiding framework for rural health research may lead to greater frequency of academic-community partnerships and facilitate these potentially meaningful changes in rural healthcare delivery. Thus, our approach will emphasize engagement with the community to increase the salience and fit of the research performed in these settings.

The CFIR (82) and iPARIHS (81) frameworks were less frequently cited, but were used effectively in the studies that employed them as determinant frameworks. The primary use of CFIR was determination of the facilitators and barriers to implementation as an initial step in the development and evaluation of the intervention to be tested. iPARIHS was used more diversely, including as an evaluation framework of outcomes or process, and as a guiding framework for the steps of implementation, with an emphasis on how the intervention could be best facilitated. PRISM has been identified as a determinant framework in prior work as well, where its emphasis on context as a means of identifying contextual influences on implementation success and approach (94). The uses of these frameworks in the reviewed literature is largely consistent with their use in research beyond the rural scope.

The synthesis of the research included in this review suggests that a research agenda that integrates the contributions of the RE-AIM framework couched within a CBPR approach to engagement and implementation process and informed by elements of the CFIR and i-PARIHS frameworks is ideal for development, evaluation, implementation, and dissemination of interventions targeting rural health. Specifically, our review pointed to the (1) need to begin with a CFIR-based analysis of facilitators and barriers, (2) identify implementation outcomes aligned with RE-AIM, and (3) develop and evaluate facilitation efforts consistent with the iPARIHS framework. All of these research practices are determined in consultation with rural community stakeholders to ensure contextual fit in line with the CBPR approach. We also prioritized the external environment and implementation and sustainability infrastructure domains of the PRISM framework to ensure sustainability from the financial, regulatory, and practical infrastructure perspectives. This is an intentional integration of selected components of these leading frameworks to form a research agenda rather than an explicit adherence to any one framework and is open to improvements and modification at later points in the research process.

Although the approach described here could also be applied to urban healthcare settings, it may have specific utility for the rural setting. Several examples from the reviewed literature highlight the importance of identifying barriers to effective implementation such as geographic isolation and distance from healthcare centers and meeting those challenges with telehealth and other forms of remote care. Others noted the importance of engaging communities using a CBPR approach given the outsider status of large healthcare systems that are not inherently within the rural community. Finally, as previously mentioned, the emphasis on reach within the underserved rural setting contributed to utilization of lay providers and other nontraditional care providers to enhance engagement with the target populations. Thus, these frameworks and their selected domains may help to identify lines of investigation specific to the rural health setting.

An additional notable observation from this scoping review was the tendency for some studies to reference a theory, model, or framework at some point in the article, but to never specify its use in the design, execution, or evaluation of the research. This occurred most often in the context of describing future directions for the research program or when situating the research within a larger body of literature focused on one or more theories, models, and frameworks. The importance of clearly specifying a theory, model, or framework is analogous to the recommendation of Proctor et al. to name, define, and operationalize implementation strategies used in dissemination and implementation research (95). As noted by those authors, the absence of specification of implementation strategies contributes to the “Tower of Babel” phenomenon in implementation science, meaning that inconsistent use and operationalization of terminology leads to confusion in the field. That confusion functions as a barrier to progress in implementation science including in understanding of the application of its own theories, models, and frameworks (96).

A failure to adequately specify theories, models, and frameworks similarly hampers efforts to improve understanding of what works in implementation science. Research teams would do well to similarly specify the theory, model, or framework used in the research process by naming it, ideally with primary source citation, and describing how it was used, particularly at the phase of research where it was applied (e.g., designing, executing, or evaluating). Such reporting could also clearly articulate which elements of a given theory, models, or framework were most emphasized, or alternatively, which elements were excluded from the work. As described in the method used for this review, we included several studies where a given theory, model, or framework was not specified in the final outcomes report, but was included in the protocol paper. This likely reflects the study teams’ tendency to apply a theory, model, or framework in the initial planning phases of the research, but not to subsequently specify and describe its use in the later work.

### 4.1 Limitations

This scoping review relied on authors’ reporting of dissemination and implementation theories, models, and frameworks for inclusion in the reviewed literature. A well-known deficit in the clinical trials and quality improvement literature is the inconsistent specification and reporting of theories, models, and frameworks (7). Therefore, this review likely does not include many studies that used one or more theory, model, or framework but did not specifically acknowledge or cite it. As previously noted, a recommendation based on this limitation is that implementation scientists more clearly specify their use of theories, models, and frameworks in methods sections, as well as how they are used and operationalized, similar to prior recommendations to specify implementation strategies (95).

Additionally, our review focused only on trials and practical implementation studies (e.g., quality improvement projects) of healthcare interventions, seeking to understand the use of theories, models, and frameworks specific to research evaluating and implementing healthcare interventions. Thus, we did not include in our review the many studies aimed at better understanding the barriers that might exist to implementing an intervention. Much of this research consists of formative evaluation that may effectively use theories, models, and frameworks to guide identification of various barriers and facilitators and subsequent modifications to the intervention. Those studies do not depict the actual deployment and evaluation of the intervention approach itself, which was the focus of this review. A subsequent review may effectively scope and summarize the formative research that is critical to advancing healthcare.

### 4.2 Conclusions

The proliferation of theories, models, and frameworks in implementation science presents numerous choices for consideration when designing research trials and quality improvement initiatives aimed at improving rural health outcomes. This review sought to examine the scope of these options and determine which theories, models, and frameworks were most prominent for developing a research agenda specific to dissemination and implementation research and practice in rural health.

From this scoping review, we specifically identified RE-AIM as the primary framework for evaluating outcomes in rural healthcare implementation research, which is consistent with the broader field. CBPR represented a particularly applicable framework for conducting healthcare implementation research in rural settings. Given its explicit focus on effectively engaging communities as partners in the research process, CBPR would likely increase the buy-in of participants and thus the generalizability of findings and overcome the tendency for research teams to “parachute in” to communities with which they are usually disconnected. CFIR and iPARIHS were recognized as the leading frameworks for the identification of determinants of implementation and opportunities to improve facilitation of implementation, respectively.

Accordingly, we concluded that a combination of the strengths drawn from RE-AIM (79), CBPR (80), iPARIHS (81), CFIR (82) has the potential to inform an approach for structuring implementation research in rural communities based on the available evidence for their utility in a variety of relevant dissemination and implementation work performed over nearly four decades of research.

## Data Availability

The original contributions presented in the study are included in the article/**Supplementary Materials**, further inquiries can be directed to the corresponding author.
