# Supplementary material for: Implementing evidence-based practices in rural settings: a scoping review of theories, models, and frameworks
Source: Front Health Serv. 2024 Jul 5;4:1326777. doi: 10.3389/frhs.2024.1326777 (PMC11258036; doi:10.3389/frhs.2024.1326777)
Supplement: Supplementary file 2 [file Table2.docx]

Additional File 2.

| Principal Investigator | Robert E. Brady |
| --- | --- |
| Librarians | Pamela J. Bagley and Elaina J. Vitale |
| Short Topic | Studies conducted in a rural setting that cite a dissemination/ implementation framework publication |
| Search Date | The search was run on Feb 2, 2022 |

**Scopus (Elsevier)**

| 3 | #1 AND #2 | [1,677 results](https://www-scopus-com.dartmouth.idm.oclc.org/search/history/results.uri?origin=searchhistory&shid=6) |
| --- | --- | --- |
| 2 | TITLE-ABS-KEY ( rural ) | [433,828 results](https://www-scopus-com.dartmouth.idm.oclc.org/search/history/results.uri?origin=searchhistory&shid=2) |
| 1 | REFTITLE ( "Bridging science to service: using Rehabilitation Research and Training Center program to ensure that research-based knowledge makes a difference" OR "Knowledge dissemination and utilization in gerontology: an organizing framework" OR "ACE Star Model of EBP: Knowledge Transformation" OR "Implementation research: a synthesis of the literature" OR "Adaptation in dissemination and implementation science" OR "Advancing adherence research in sport injury prevention" OR "Advancing health disparities research within the health care system: a conceptual framework" OR "The ARC organizational and community intervention strategy for implementing evidence-based children's mental health treatments" OR "Randomized trial of MST and ARC in a two-level evidence-based treatment implementation strategy" OR "The behaviour change wheel: A new method for characterising and designing behaviour change interventions" OR "The Behaviour Change Wheel: A Guide To Designing Interventions" OR "Blueprint for the dissemination of evidence-based practices in health care" OR "Achieving evidence-based nursing practice: Impact of the Caledonian Development Model" OR "The role and theoretical evolution of knowledge translation and exchange in public health" OR "The Institute of Musculoskeletal Health and Arthritis (IMHA) knowledge exchange task force: An innovative approach to knowledge translation" OR "Lost in knowledge translation: Time for a map" OR "The diffusion of effective behavioral interventions project: development, implementation, and lessons learned" OR "Linking research and practice: evidence-based HIV prevention" OR "Replicating effective programs: HIV/AIDS prevention technology transfer" OR "The HIV/AIDS Prevention Research Synthesis Project: scope, methods, and study classification results" OR "Strengthening HIV prevention: application of a research-to-practice framework" OR "De-implementing wisely: developing the evidence base to reduce low-value care" OR "An Idea Worth Sustaining: Evaluation of the sustainability potential of Choosing Wisely across Ontario Community Hospitals and Family Health Teams" OR "Pursuing common agendas: a collaborative model for knowledge translation between research and practice in clinical settings" OR "Introduction to methods in community-based participatory research for health" OR "REVIEW OF COMMUNITY-BASED RESEARCH: Assessing Partnership Approaches to Improve Public Health" OR "Implementation research design: integrating participatory action research into randomized controlled trials" OR "Sustained, intensive engagement to promote health and safety knowledge transfer to and utilization by workplaces" OR "A conceptual framework for the comparative analysis of policy change: Measurement, explanation and strategies of policy dismantling" OR "Implementation research in mental health services: an emerging science with conceptual, methodological, and training challenges" OR "The utilization of policy analysis by state agency officials" OR "Can the Canadian Heart Health Initiative inform the population Health Intervention Research Initiative for Canada" OR "Conceptualizing dissemination research and activity: the case of the Canadian Heart Health Initiative" OR "Using research to improve nursing practice: A guide CURN project" OR "The ConNECT Framework: a model for advancing behavioral medicine science and practice to foster health equity" OR "Fostering implementation of health services research findings into practice: a consolidated framework for advancing implementation science" OR "Contextual Frameworks for Research on the Implementation of Complex System Interventions" OR "Social marketing and diffusion-based strategies for communicating with unique populations: HIV prevention in San Francisco" OR "A convergent diffusion and social marketing approach for disseminating proven approaches to physical activity promotion" OR "Retailing research: increasing the role of evidence in clinical services for childbirth" OR "Mapping new theoretical and methodological terrain for knowledge translation: contributions from critical realism and the arts" OR "The case for knowledge translation: shortening the journey from evidence to effect" OR "The awareness-to-adherence model of the steps to clinical guideline compliance: the case of pediatric vaccine recommendations" OR "Health program planning: an educational and ecological approach" OR "Designing and evaluating interventions to eliminate racial and ethnic disparities in health care" OR "Determinants of innovation within health care organizations: Literature review and Delphi study" OR {Diffusion of innovations} OR "Development of a Dissemination and Implementation Framework for an Early Childhood Obesity Prevention Program" OR "Contextual factors influencing readiness for dissemination of obesity prevention programs and policies" OR "Introduction to the special section on dissemination: Dissemination research and research dissemination: How can we close the gap" OR "Knowledge translation versus knowledge integration: A funder's perspective" OR "Translating research into improved outcomes in comprehensive cancer control" OR "The dynamic sustainability framework: addressing the paradox of sustainment amid ongoing change" OR "Effective dissemination strategies" OR "Knowledge transfer and exchange frameworks in health and their applicability to palliative care: scoping review protocol" OR "Advancing health equity through CTSA programs: Opportunities for interaction between health equity, dissemination and implementation, and translational science" OR "An Evidence Integration Triangle for Aligning Science with Policy and Practice" OR "Making psychological theory useful for implementing evidence based practice: A consensus approach" OR "Advancing a conceptual model of evidence-based practice implementation in public service sectors" OR "Systematic review of the Exploration, Preparation, Implementation, Sustainment (EPIS) framework" OR "Facilitation of Best Practices (FAB) Framework" OR "Integration of targeted health interventions into health systems: a conceptual framework for analysis" OR "Diffusion of complex health innovations--implementation of primary health care reforms in Bosnia and Herzegovina" OR "Policy dissemination research" OR "A Framework for Enhancing the Value of Research for Dissemination and Implementation Research" OR "Development of a framework for knowledge translation: understanding user context" OR "The improvement guide: a practical approach to enhancing organizational performance" OR "Using a framework for spread: the case of patient access in the Veterans Health Administration" OR {Public health model} OR "A framework for the dissemination and utilization of research for health-care policy and practice" OR "From science to service: a framework for the transfer of patient safety" OR "Obesity prevention: A proposed framework for translating evidence into action" OR "Interventions in organizational and community context: a framework for building evidence on dissemination and implementation in health services research" OR "Towards a general theory of implementation" OR "Generic Implementation Framework (GIF)" OR "Diffusion of Innovations in Service Organizations: Systematic Review and Recommendations" OR "The health equity implementation framework: proposal and preliminary study of hepatitis C virus treatment" OR "A framework for disseminating evidence-based health promotion practices" OR "Health promotion technology transfer: organizational perspectives" OR "Implementing computerized technology: an organizational analysis" OR "The challenge of innovation implementation" OR "Interacting elements of integrating science, policy, and practice" OR "Bridging the gap between prevention research and practice: the interactive systems framework for dissemination and implementation" OR "Intervention mapping: a process for developing theory- and evidence-based health education programs" OR "The Iowa model of evidence-based practice to promote quality care" OR "Johns Hopkins nursing evidence based practice model and guidelines" OR "The Joint Venture Model of Knowledge Utilization: A Guide for Change in Nursing" OR "Knowledge brokering: exploring the process of transferring knowledge into action" OR "Developing a framework for transferring knowledge into action: a thematic analysis of the literature" OR "Exploring knowledge exchange: a useful framework for practice and policy" OR "Knowledge transfer and exchange: Review and synthesis of the literature" OR "How should we assess knowledge translation in research organizations; designing a knowledge translation self-assessment tool for research institutes (SATORI)" OR "Knowledge translation for research utilization: design of a knowledge translation model at Tehran University of Medical Sciences" OR "Using linking systems to build capacity and enhance dissemination in heart health promotion: a Canadian multiple-case study" OR "Enhancing dissemination through marketing and distribution systems: a vision for public health" OR "A model for improving the dissemination of nursing research" OR "The use of research in local health service agencies" OR "Innovation adoption: A review of theories and constructs" OR "Organizational innovation adoption: a multi-level framework of determinants and opportunities for future research" OR "Normalization process theory on-line users' manual and toolkit" OR "Implementing, embedding, and integrating practices: an outline of normalization process theory" OR "Normalisation process theory: a framework for developing, evaluating and implementing complex interventions" OR "Using organization theory to understand the determinants of effective implementation of worksite health promotion programs" OR "Toward a comprehensive interdisciplinary model of health care research use" OR "The Ottawa Model of Research Use" OR "Outcomes-focused knowledge translation: A framework for knowledge translation and patient outcomes improvement" OR "A social marketing model for disseminating research-based treatments to addictions treatment providers" OR "Pathways to evidence-informed policy and practice: a framework for action" OR "Evidence-based approaches to dissemination and diffusion of physical activity interventions" OR "A practical, robust implementation and sustainability model (PRISM) for integrating research findings into practice" OR "The PRECIS-2 tool: designing trials that are fit for purpose" OR "A pragmatic-explanatory continuum indicator summary (PRECIS): a tool to help trial designers" OR "Making clinical trials more relevant: improving and validating the PRECIS tool for matching trial design decisions to trial purpose" OR "Health program planning: an educational and ecological approach" OR "Outcomes for Implementation Research: Conceptual Distinctions, Measurement Challenges, and Research Agenda" OR "Enabling the implementation of evidence based practice: a conceptual framework" OR "Evaluating the successful implementation of evidence into practice using the PARiHS framework: theoretical and practical challenges" OR "The PARIHS framework--a framework for guiding the implementation of evidence-based practice" OR "Translating evidence into practice: a model for large scale knowledge translation" OR "Inferring strategies for disseminating physical activity policies, programs, and practices from the successes of tobacco control" OR "Introduction to the special section on dissemination: Dissemination research and research dissemination: How can we close the gap" OR "Knowledge translation versus knowledge integration: A finder's perspective" OR "Translating research into improved outcomes in comprehensive cancer control" OR "Persuasive communication and medical technology assessment" OR "Evaluating the public health impact of health promotion interventions: the RE-AIM framework" OR "RE-AIM Planning and Evaluation Framework: Adapting to New Science and Practice With a 20-Year Review" OR "Leading clinical practice change" OR "Shaping strategic change: making change in large organizations: the case of the National Health Service" OR "Implementing evidence-based interventions in health care: application of the replicating effective programs framework" OR "Planning for innovation through dissemination and utilization of knowledge" OR "Determining research knowledge infrastructure for healthcare systems: a qualitative study" OR "Institute for Work and Health--Knowledge Transfer & Exchange Guides" OR "Assessing country-level efforts to link research to action" OR "How can research organizations more effectively transfer research knowledge to decision makers" OR "A model for change to evidence-based practice" OR "Dissemination of physical activity evidence, programs, policies, and surveillance in the international public health arena" OR "Introduction and Conceptual Model for Utilization of Prevention Research" OR "A Framework for Research Utilization Applied to Seven Case Studies" OR "Updating the Stetler Model of Research Utilization to Facilitate Evidence-Based Practice" OR "Sticky knowledge: a possible model for investigating implementation in healthcare contexts" OR "Exploring internal stickiness: impediments to the transfer of best practice within the firm" OR "Development of a framework and coding system for modifications and adaptations of evidence-based interventions" OR "Agendas, alternatives, and public policies" OR "A technology transfer model for effective HIV/AIDS interventions: Science and practice" OR "Developing theory-informed behaviour change interventions to implement evidence into practice: a systematic approach using the Theoretical Domains Framework" OR "Validation of the theoretical domains framework for use in behaviour change and implementation research" OR "A guide to using the Theoretical Domains Framework of behaviour change to investigate implementation problems" OR "Transcreation: an implementation science framework for community-engaged behavioral interventions to reduce health disparities" OR "A translational framework for public health research" OR "The role of translational research in addressing health disparities: A conceptual framework" OR "Quality Enhancement Research Initiative (QUERI): A collaboration between research and clinical practice" OR "Overview of the Veterans Health Administration (VHA) Quality Enhancement Research Initiative (QUERI)" OR "Lessons learned about implementing research evidence into clinical practice" OR "Diffusion theory and knowledge dissemination, utilization, and integration in public health" OR "A conceptual model for growing evidence-based practice" OR "A theory of organizational readiness for change" ) | [58,311 results](https://www-scopus-com.dartmouth.idm.oclc.org/search/history/results.uri?origin=searchhistory&shid=1) |
